# Neighboring and Connectivity-Aware Routing in VANETs

**DOI:** 10.1155/2014/789247

**Published:** 2014-05-21

**Authors:** Huma Ghafoor, Insoo Koo, Nasir-ud-Din Gohar

**Affiliations:** ^1^The School of Electrical Engineering, University of Ulsan, San 29, Muger 2-dong, Ulsan 680-749, Republic of Korea; ^2^School of Electrical Engineering and Computer Science, National University of Sciences and Technology (NUST), H-12, Islamabad 44000, Pakistan

## Abstract

A novel position-based routing protocol anchor-based connectivity-aware routing (ACAR) for vehicular ad hoc networks (VANETs) is proposed in this paper to ensure connectivity of routes with more successfully delivered packets. Both buses and cars are considered as vehicular nodes running in both clockwise and anticlockwise directions in a city scenario. Both directions are taken into account for faster communication. ACAR is a hybrid protocol, using both the greedy forwarding approach and the store-carry-and-forward approach to minimize the packet drop rate on the basis of certain assumptions. Our solution to situations that occur when the network is sparse and when any (source or intermediate) node has left its initial position makes this protocol different from those existing in the literature. We consider only vehicle-to-vehicle (V2V) communication in which both the source and destination nodes are moving vehicles. Also, no road-side units are considered. Finally, we compare our protocol with A-STAR (a plausible connectivity-aware routing protocol for city environments), and simulation results in NS-2 show improvement in the number of packets delivered to the destination using fewer hops. Also, we show that ACAR has more successfully-delivered long-distance packets with reasonable packet delay than A-STAR.

## 1. Introduction

Vehicular ad hoc network (VANET) is a promising means for safe driving by enabling cooperation among vehicles. Traffic accidents are a serious issue all over the world. Millions of deaths are caused by road accidents. Researchers have already implemented and are attempting to implement various safety applications, both in academia and industry.

Vehicular ad hoc network (VANET), an up- and-coming technology, is a combination of an ad hoc network, wireless LAN, and cellular technology [1]. It is a technique in which wireless technology is deployed in vehicles. Each vehicle acts as a node that can potentially forward data packets towards the destination, thereby forming an ad hoc network in which nodes can join and depart in a dynamic manner [2]. It is also known as intervehicle communication (IVC) or vehicle-to-vehicle (V2V) communication [3]. VANETs have become popular due to their vast range of applications. Applications for purposes other than safety are given in [3–7].

Routing in VANET is a current area of research, both in academia and industry. VANET is a subclass of mobile ad hoc network (MANET) in which nodes are vehicles, so their nature is dynamic. Due to the highly dynamic nature of the nodes, efficient routing is a key challenge. So, there is need for a routing protocol which provides better information delivery without route breakage. Routing protocols for VANETs are classified into five main categories [3]: ad hoc/topology-based, position-based/geographic, cluster-based, broadcast, and geocast routing. Among these categories, position-based and geocast routing protocols are best suited [2] for VANETs, as other types of protocols have delay and overhead problems. Geocast routing is used for the delivery of packets from the source to an exact geographic region. We consider unicast routing, that is, from a single source to a single destination, in our paper. Therefore, we discuss only position-based routing in this paper. Other protocols are discussed in [2].

In this paper, a novel position-based routing protocol, ACAR [1], is proposed for use in the city. It considers both buses and cars as vehicular nodes moving in both clockwise and anticlockwise directions. Buses are defined as city buses running according to predefined routes. As in previous research, junctions are also called “anchors,” where the decision is taken. The decision is the selection of neighboring node to trace the source or destination node. This is a hybrid protocol in which both the greedy forwarding approach and the store-carry-and-forward approach are used. We consider two scenarios: when the network is fully connected and when the network is sparse. We then compare it with A-STAR [8] using five metrics. A-STAR is a plausible protocol which does not consider static destinations but does consider the connectivity of routes. Our comparison shows that the performance of ACAR is better.

The remaining paper is organized as follows. Related work is presented in Section 2. In Section 3, ACAR is described.  Section 4 presents the performance results, while Section 5 provides our concluding remarks.

## 2. Related Work

Position-based routing depends on the geographic position of the destination. It is mainly proposed for ad hoc networks and does not use network addresses to send a message from source to destination. Position-based routing is broadly divided into three main categories [9]:

1. nondelay tolerant network (non-DTN);
2. delay tolerant network (DTN);
3. hybrid.

### 2.1. Nondelay Tolerant Network

Protocols in this category are based on greedy forwarding. In greedy forwarding, each node requires three pieces of information: its own position, the positions of its neighbors, and the position of the destination. We do not describe here how greedy forwarding is applied in any scenario. An example can be seen in [1]. In this subsection, we briefly discuss the existing protocols in this category and describe their drawbacks.

Greedy perimeter stateless routing (GPSR) [10] is a well-known routing protocol that uses greedy forwarding. Due to its recovery approach, problems like routing loops, the creation of longer paths, and incorrect packet directions are encountered when using GPSR in city scenarios. Also, GPSR has a low packet delivery ratio and was proposed for MANETs. Greedy perimeter routing with lifetime (GPSR-L) [11] and vehicular routing protocol (VRP) [12] are modified versions of GPSR proposed for VANETs, which have improved packet delivery ratios. In VRP, road junctions and packet delay are not considered. Another protocol, called GPSR J+, considers road junctions and was proposed in [13]. It is a prediction-based protocol which also has a low packet delivery ratio.

Anchor-based street and traffic aware routing (A-STAR) [8] is a connectivity-aware routing protocol which uses city bus route information. The algorithm finds the shortest path by considering the connectivity of vehicular nodes. Here, weights are assigned on the basis of traffic density and then, using Dijkstra's algorithm, the shortest path is selected. This algorithm becomes stuck in a local maximum when there is no neighboring node that is closer to the destination. Landmark overlays for urban vehicular routing environments (LOUVR ) [14], road-based using vehicular traffic-reactive routing (RBVT-R), road-based using vehicular traffic-proactive routing (RBVT-P) [15], edge node based greedy routing (EBGR) [16], and border-node based most forward within radius routing (B-MFR) [17] are other existing protocols for VANETs in this category. Their packet delivery ratios are shown in Table 1. EBGR and BMFR have very low delivery ratios.

### 2.2. Delay Tolerant Network

Delay tolerant network, also called disruption tolerant network [18], is based on the store-carry-and-forward principle. These protocols are used in scenarios when there is no neighbor node in the network, that is, when the network is sparse. In these protocols, data is delivered from vehicles to static infrastructure access points. This approach is helpful in our protocol when the greedy forwarding approach fails. In the store-carry-and-forward approach, a packet is stored when there is no neighboring node in the network. As any node comes into the communication range of the relevant node, the packet is carried until the node forwards it, either to a node moving in the direction of destination or to the destination node itself.

Most of the existing protocols for VANETs in this category were proposed for static destinations. Vehicle-assisted data delivery (VADD) [4] is based on a carry-and-forward scheme. This algorithm considers sparse network conditions but was proposed for static destinations. Shafiee et al. [5] proposed a protocol connectivity-aware minimum delay geographic routing (CMGR) using the same carry-and-forward scheme to deliver packets from a moving vehicle to a fixed destination. They compared CMGR with VADD and showed that CMGR performs better. Similarly, other routing protocols in this category are static-node assisted adaptive routing protocol in vehicular networks (SADV) [19], delay-bounded greedy forwarding (D-greedy), and delay-bounded min-cost forwarding (D-MinCost) [20].

### 2.3. Hybrid

Hybrid is a combination of a nondelay tolerant network and a delay tolerant network. GeoDTN+Nav [21] is an example of a hybrid protocol. When the flow of traffic is at its peak, that is, the network is fully connected, the nondelay tolerant network approach is used. On the other hand, when the network is sparse, the protocol is switched to the delay tolerant network approach, that is, a store-carry-and-forward scheme. Also, in this paper, the authors assume that the destination is static. Switching from one mode to another can cause delay because, in GeoDTN+Nav, packet first switches to perimeter mode before moving to the delay tolerant network approach.

A comparison of all the routing protocols on the basis of delivery ratios and latency, as discussed in the previous three subsections, is given in Table 1.

All existing protocols for VANETs in the literature have only one basic issue, that is, path connectivity. As the nodes are moving vehicles, it is difficult to maintain a stable path. Existing routing protocols perform better in highway scenarios but, when deployed in city scenarios, their performance is degraded. Greedy forwarding is the core of the routing used in VANETs, but there is no valid solution when greedy forwarding fails, especially in V2V communication. To the best of our knowledge, to ensure successful packet delivery in city scenarios, existing protocols consider a fixed unit. In some protocols, a fixed destination is used to ensure packet delivery, while others have roadside units that help in sparse network conditions, thus improving the success rates of their protocols. Our goal is for communication to be entirely V2V in a city scenario, without using any fixed unit.

## 3. Anchor-Based Connectivity-Aware Routing (ACAR)

In this section, we explain the proposed ACAR protocol. ACAR is a hybrid protocol which, in a city scenario, uses both the greedy forwarding approach and the store-carry-and-forward approach to provide a better packet delivery ratio with acceptable delay and overhead. The purpose of using greedy forwarding is the same as in all existing VANET protocols. ACAR reduces the number of hops by selecting the neighboring node closest to the destination, thus making routing more efficient. The basic goal is to find a solution when greedy forwarding fails in a city scenario. To solve this problem, we used a store-carry-and-forward approach that helps our protocol in sparse conditions.

ACAR is a connectivity-aware routing protocol. As the name implies, it is a routing protocol that finds routes by considering the connectivity of vehicular nodes. The source and destination are both moving nodes. To verify our idea, we compared our protocol with an existing routing protocol, A-STAR, using five metrics, and showed that our protocol performed better in all cases. A-STAR is a plausible connectivity-aware routing protocol that considers both the source and destination as moving vehicular nodes. The following assumptions are used in this protocol.

### 3.1. Assumptions

Vehicles are assumed to have an on-board navigation system. GPS receivers and preloaded street maps are assumed to be installed on each vehicle. GPS receivers determine position and direction, which is helpful for nodes in calculating vehicle density. The number and sequence of anchors, along with bus route information, are provided by street maps. We also assume that buses are moving normally on their fixed routes with constant speed.

### 3.2. Description of Algorithm

**Figure 1:**
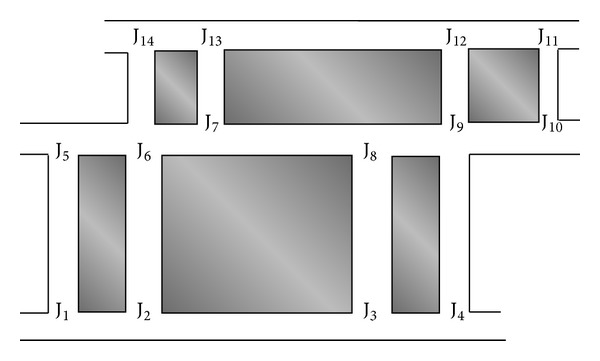
City model.

Considering both the buses and cars moving in both clockwise and anticlockwise directions, we performed our simulation in an example city with 14 anchor points (Figure 1). The anchors were the street junctions, as in previous research, and are represented by J_1_, J_2_, and so on. We again emphasize here that in this study, no roadside units were considered, and both the source and destination were moving vehicles. Therefore, this study is valid only for V2V communication. We consider only two cases: when the network is fully connected and when the network is sparse. In the following section, we discuss these two cases in which the greedy forwarding approach and the store-carry-and-forward approach are used.

*Case  1 (when the network is fully connected).* To initiate routing, a* route request beacon message (*RRBM) is sent by the source node to its closest neighbor (either moving in the direction of the destination or moving opposite to the destination) using the greedy forwarding approach. RRBM includes four items: node ID, destination ID, time, and velocity. Note that there is no need to exchange information on the position and direction of the nodes. This information is obtained by GPS and in greedy forwarding, and each node has to exchange this information with its neighboring nodes. Therefore, each node already knows the positions and directions of itself and its neighbors. The RRBM passes through several nodes then reaches the destination. The destination node sends back the* route reply message (*RRM) containing additional information, that is, the list of anchors used in the routing path. Each message has its lifetime value. Hence, a path is formed using the greedy forwarding approach in V2V communication. The packet is then transferred from source to destination using this shortest-distance path. As all nodes are moving, these RRBM and RRM are continuously broadcasted by the nodes to maintain the connectivity of the best shortest path.

**Figure 2:**
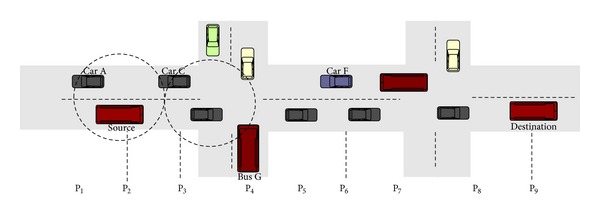
An example illustrating routing for time* t*_1_ when network is fully connected.

Let us assume that some vehicles are moving in both directions as shown by Figure 2. Figure 2 shows the positions of all moving nodes at time* t*_1_, when the RRBM is generated by the source node. With the help of this figure, we can better explain how a packet is forwarded from the source node to the destination node considering both the buses and cars moving in both clockwise and anticlockwise directions. The source node, which is a bus moving on its city route, wants to communicate to a destination node which is also a bus. The communication range of the nodes is shown by a dotted circle. In the circle, Car C is nearer to the destination than Car A. Car C receives the packet from the source node and forwards it to Bus G, which transfers it to Car F. By doing so, the packet will reach the destination using the best possible route.

**Figure 3:**
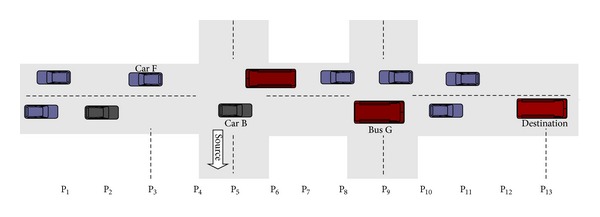
An example illustrating the scenario for time* t*_2_ when any node has left its initial position.

**Figure 4:**
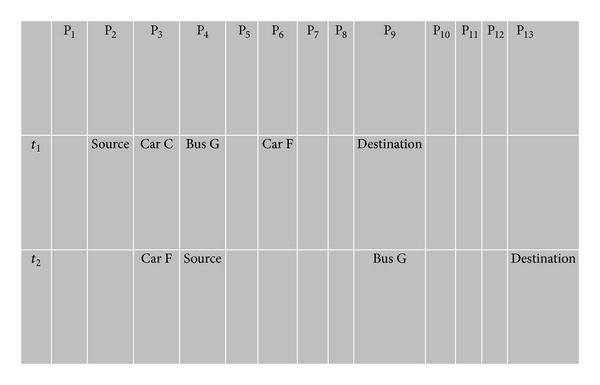
Timing diagram showing new position of nodes.

There is the possibility that any node (source or intermediate) has left its initial position before receiving the RRM from the destination node. Suppose that the source bus sends an RRBM at the position shown in Figure 2. To make the picture understandable, assume that all nodes in Figure 2 are at time* t*_1_ as shown in the timing diagram for Figure 4. When the destination bus receives the RRBM and wants to send back an RRM, the source node has left its position (refer to Figure 4; time* t*_2_ shows new position P_4_) and enters another street as shown in Figure 3. We propose that each node, before leaving the anchor point, sends a beacon message to all neighboring nodes (either car or bus) at the junction to update its new position. Although buses move on fixed routes, this might not be the case when an emergency occurs and the bus has to change its route. Hence, the information is retained at each junction. In our scenario, we consider the bus to be a source node. This scenario is applicable when the source or destination is a car. As shown in Figure 3, Car B knows the next position of the source bus. It receives the RRM and sends it to the source bus.

**Figure 5:**
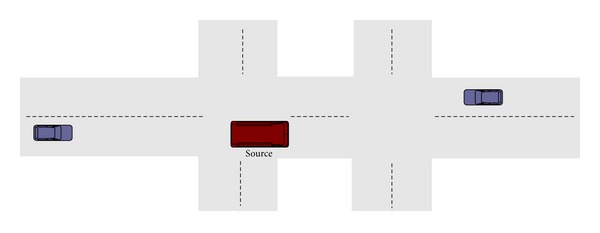
Sparse network.

*Case 2 (when the network is sparse).* In this subsection, we consider the case when the network is sparse; that is, there is no neighboring node in the range of the node which wants connectivity. That node may be the source node itself or any intermediate nodes. In this case, greedy forwarding fails and we apply the store-carry-and-forward approach [18].  Figure 5 shows this scenario. Our goal is to ensure packet delivery with acceptable delay and overhead. We propose that the node uses the store-carry-and-forward approach. We know that buses follow streets that are busier than other streets. And we also know that on busier streets, we may find some cars. Therefore, any node that encounters sparse conditions should keep the packet until any other node comes into its communication range. A delay may occur with this approach, but it is more acceptable than dropping the packet.

The pseudocode for ACAR is described in Algorithm 1.

## 4. Performance Evaluation

ACAR was simulated in network simulator-2 (NS-2) [22] using the example city scenario shown in Figure 1. In this scenario, the buses moved normally at an average speed of 50 km/h and the cars moved with various speeds from 50 km/h to 100 km/h. The total number of buses was 37, running on their fixed routes. The total area of simulation was 2000 ∗ 2000 square meters. The total number of intersections, also called anchors, was 14. The number of nodes (vehicles) was varied from 100 to 400, each having a transmission range of 200 m. We used 20 CBR connections and the size of the data packet was 64 bytes. The total simulation time was 850 seconds. Gnuplot was used to acquire the graphs. Five metrics were used to evaluate the performance of ACAR:

- packet delivery ratio (PDR);
- packet delay;
- route length distribution;
- routing overhead;
- average hop-count.

**Figure 6:**
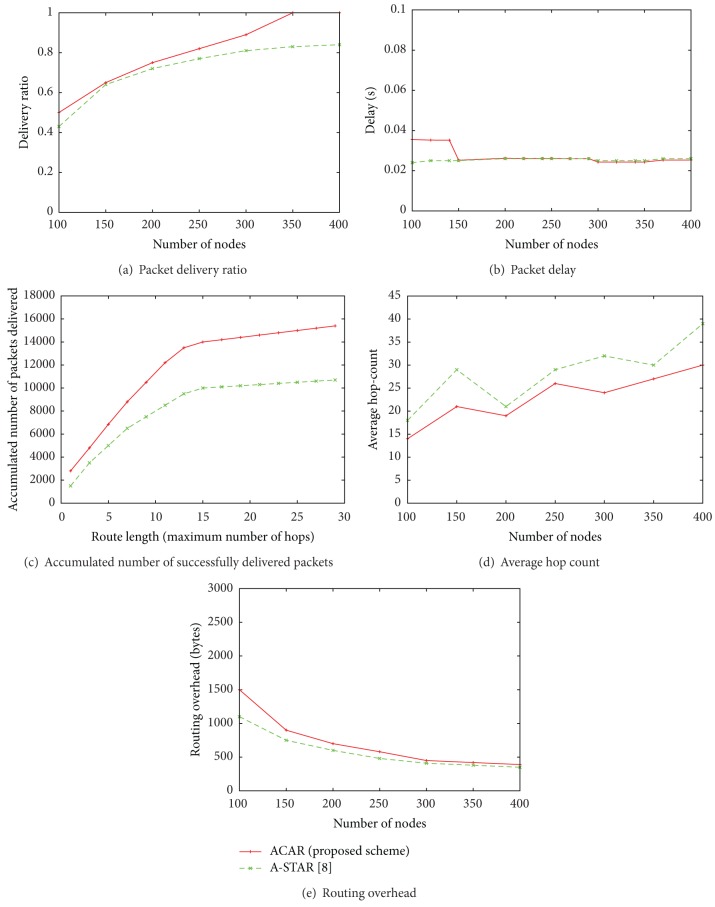
Performance comparison between ACAR (the proposed scheme) and A-STAR according to the number of nodes in terms of packet delivery ratio, packet delay, accumulated number of successfully-delivered packets, average hop-count, and routing overhead when the node vehicle's speed is 50 km/h, the radio range of node is 200 m, and maximum number of hops is 30.

Figure 6 shows the performance of both protocols according to the number of nodes when node vehicle's speed is fixed at 50 km/h. The radio range of vehicle is 200 m and maximum number of hops to the destination is 30. The packet delivery ratio of ACAR and A-STAR for an example city scenario (see Figure 1) is shown in Figure 6(a). The packet delivery ratio is the ratio of the number of packets delivered to the destination to the number generated by the source node. We take the number of nodes as an independent variable. As the number of nodes increased, the packet delivery ratio increased since connectivity improved. The ACAR provides 100% packet delivery when there were more than 350 vehicles, that is, when the network was fully connected. On the other hand, when the network was sparse, the packet delivery ratio of the ACAR is degraded due to connectivity problems. For example, the ACAR provides only 50% packet delivery when the number of nodes is 100. However, Figure 6(a) shows that the ACAR provides better packet delivery ratio than A-STAR regardless of the number of nodes. The degradation in the packet delivery ratio during sparse network conditions also affected packet delay.  Figure 6(b) shows that the proposed scheme provides more delay than A-STAR when number of nodes is less than 150. The reason for the delay was that the ACAR utilizes the store-carry-and-forward approach. Therefore, in sparse network condition, the ACAR would have more possibility that each packet had to wait until a vehicle reached the relevant node than A-STAR. However, as the number of nodes increased, both protocols had similar packet delay. As can be seen in Figure 6(b), the ACAR provides slightly better delay performance than A-STAR when the number of nodes is more than 300. Hence, we can say that as the node number increased, the delay performance of ACAR improved. It is also noteworthy that there was no linear relationship between number of nodes and packet delay in both schemes. Evaluating these metrics, we concluded that connectivity is an important issue in V2V communication.

Figure 6(c) depicts the accumulated number of successfully delivered packets of both ACAR and A-STAR. We can see that packet delivery was more successful when the network was fully connected. In this figure, the independent variable is route length, which shows the distance travelled by the packets, that is, the maximum number of hops from the source to the destination. The evaluation of this metric also describes one of the reasons for packet delays. As shown in the graph that when the network is sparse, there were more chances for the packets to drop. But, having the same number of hops for both protocols, the dropping rate of packets in A-STAR was high. Hence, we had more packets successfully delivered over long distances. These packets are dropped if the store-carry-and-forward approach is not used.  Figure 6(d) shows the average number of hops that each packet travelled from source to destination for both ACAR and A-STAR. The packets of ACAR required fewer hops compared to A-STAR because of the assumptions we made about the position and direction of each vehicular node.  Figure 6(e) shows the routing overhead of both routing protocols. The routing overhead is defined as the number of control messages transmitted by each vehicle to neighboring vehicles to maintain a route. ACAR had a slightly higher routing overhead than A-STAR because, in the proposed protocol, vehicles update each other with their position continuously to maintain connectivity. Almost all position-based routing protocols have the same overhead. The only difference is due to the number of control/beacon messages used in this scenario. From the figure, it can be seen that, as the number of vehicles increased, both protocols had similar overhead. When vehicle density increased, successful delivery of packets increased, and therefore routing failures decreased, which decreases the overhead. When the density was low, there were more chances for failed packet delivery. In our scenario, to maintain the connectivity of the routes, more beacon messages were transmitted than in A-STAR. This cost is acceptable because it allows for a delivery ratio of 100% with reasonable end-to-end delay.

**Figure 7:**
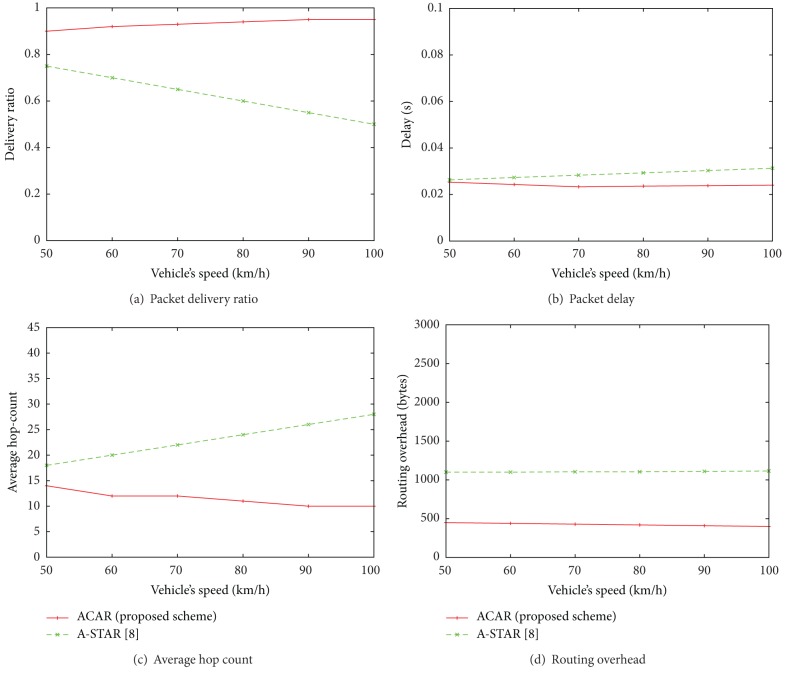
Performance comparison between ACAR (the proposed scheme) and A-STAR according to vehicle's speed in terms of packet delivery ratio, packet delay, average hop-count, and routing overhead when the number of nodes is 100 and the radio range of node is 200 m.

Figure 7 shows the evaluation of ACAR and A-STAR according to the vehicle's speed. Our purposed scheme is to make the stable routing path in sparse condition. Also, we assume that buses are running on their fixed route with constant speed. In our scenario, both source and destination nodes are buses. Therefore, when the network is sparse, the performance becomes better with varying vehicle's speed. Due to store-carry-and-forward approach, any neighbouring vehicle reaches the destination in short time which reduces the delay. Also, the number of hops and overhead is reduced in our algorithm. But, the performance of A-STAR is degraded because in sparse network a new path is required to be calculated every time.

**Figure 8:**
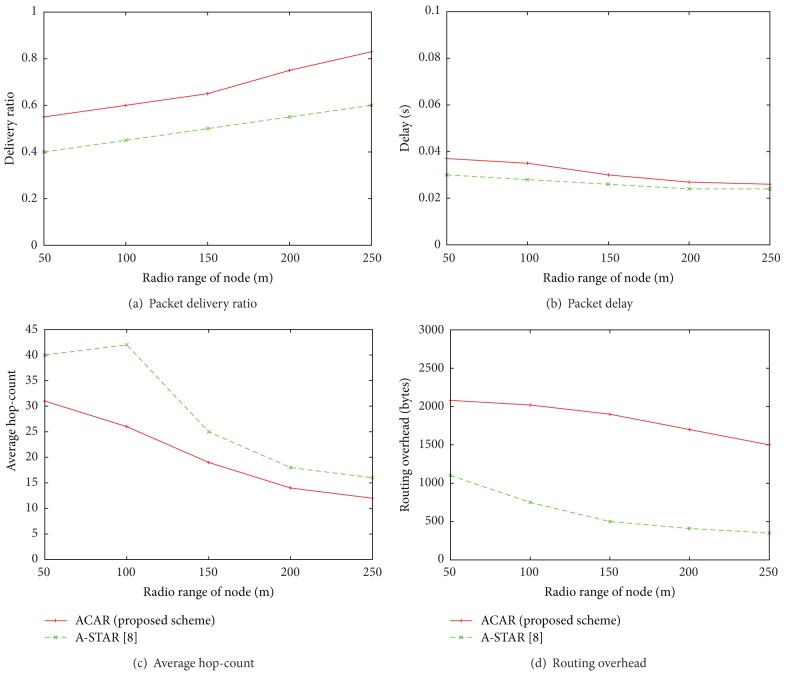
Performance comparison between ACAR (the proposed scheme) and A-STAR according to the radio range of node in terms of packet delivery ratio, packet delay, average hop-count, and routing overhead when the node vehicle's speed is 50 km/h and the number of nodes is 100.

Figure 8 shows the performance of both ACAR and A-STAR according to the radio range of node when node vehicle's speed is 50 km/h and the number of nodes is 100. The performance of all parameters improves as the radio range of node is increased. However, with small radio range, it is hard to find any neighbor in one's range. Therefore, the network becomes sparse and delay is increased. Also, the overhead is increased because nodes need to continuously update their neighboring information with each other to maintain the connectivity. This overhead of Figure 8(d) is different with that of Figures 6(e) and 7(d) because the number of nodes is small and the radio range is also varying, which makes routing failures be increased. Therefore, Figure 8(d) shows that ACAR provides larger overhead than A-STAR.  Figure 6(e) shows that the overhead of ACAR is slightly higher than A-STAR only when the network is sparse, that is, when number of nodes is less than 300.  Figure 7(d) shows that the overhead of ACAR is better than that of A-STAR when vehicle's speed increases since the destination is reached in short time which reduces the number of control/beacon messages. In summary, it is observed that overhead is increased when the network is sparse, that is, when number of nodes is small or the radio range of node is small. Also, the overhead is decreased when the speed of vehicles increases. However, Figures 8(a) and 8(c) show that in ACAR we still have better delivery ratio and less number of hops than A-STAR because of the assumptions we made about the position and direction of each vehicular node in the proposed protocol.

## 5. Conclusion

In this paper, a novel position-based routing protocol, ACAR, is proposed for city scenarios. The exploitation of both the greedy approach and the store-carry-and-forward approach in V2V communication makes this protocol unique from existing protocols. Our comparison shows a better packet delivery ratio with a higher number of successfully-delivered packets, reasonable delay, and a lower hop-count, while requiring little overhead. In the future, we will consider signal points at the junctions to reduce delay and overhead. This work is valid only when city buses are running. We will also extend our work for use in night traffic.

## Figures and Tables

**Algorithm 1:**
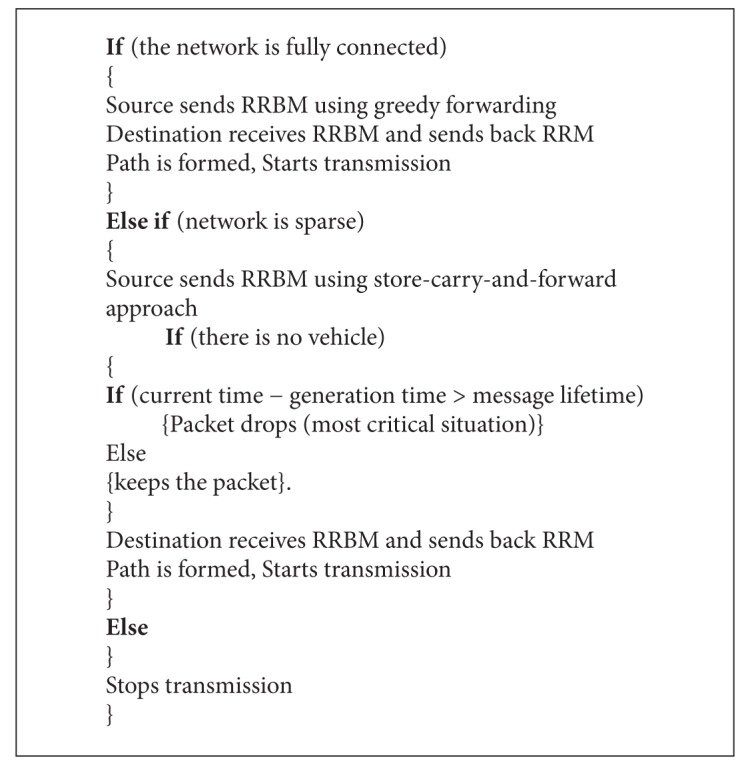


**Table 1:** Comparison of routing protocols.

Routing protocols	Type of routing	Delivery ratio	Latency (sec)	Environment applicable
GPSR	Non-DTN	Low	High*	Urban
GPSR-L	Non-DTN	Better	ND	Highway
VRP	Non-DTN	High	ND	Highway
GPSR J+	Non-DTN	Low	High*	Urban
GRANT	Non-DTN	Low	ND	Urban
A-STAR	Non-DTN	High	High*	Urban
LOUVRE	Non-DTN	Better	High*	Urban
RBVT-R	Non-DTN	High	High*	Urban
RBVT-P	Non-DTN	Better	Low*	Urban
EBGR	Non-DTN	Low	Low*	Urban and Highway
B-MFR	Non-DTN	Low	High*	Urban
VADD	DTN	High	Low*	Urban
CMGR	DTN	High	High*	Urban
SADV	DTN	Low	High*	Urban and highway
D-Greedy	DTN	High	High*	Urban
D-MinCost	DTN	High	High*	Urban
GeoDTN + Nav	Hybrid	High	High*	Urban

Low: below 50%; better: between 50% and 70%; high: above 70%.

Low*: below 0.04; high*: above 0.04; ND: not determined.
